# Editorial: Stress, anxiety, and the synapse

**DOI:** 10.3389/fnbeh.2022.1085850

**Published:** 2022-11-21

**Authors:** Kristin L. Gosselink, Jorge A. Sierra Fonseca, Daniela Jezova

**Affiliations:** ^1^Department of Physiology and Pathology, Burrell College of Osteopathic Medicine, Las Cruces, NM, United States; ^2^Department of Science, Chatham University, Pittsburgh, PA, United States; ^3^Biomedical Research Center, Institute of Experimental Endocrinology, Slovak Academy of Sciences, Bratislava, Slovakia

**Keywords:** glutamate, AMPA, amygdala, nucleus accumbens, medial prefrontal cortex, bed nucleus of stria terminalis (BNST)

Anxiety is a normal emotional or adaptive response to situations that induce fear or unease in an individual. Feelings of enhanced anxiety have increased globally as a result of the recent COVID-19 pandemic (Penninx et al., 2022). Elevations in perceived stress are implicated in this post-pandemic decline in mental health, but individual differences have also been observed in stress responsivity and resilience (Venanzi et al., 2022). The stress response aids the mind and body in overcoming harmful events, but negative consequences can occur if coping strategies are inappropriate or the exposure to stress stimuli is frequent or chronic (Jezova and Herman, 2020). In the relationship between stress and mental disorders, neuroplasticity plays a critical role and synaptic plasticity is an important component of this process. Several neurotransmitters are involved in the mechanisms of synaptic plasticity, but the contributions of glutamate and its receptors are unequivocal. Given that the relationship between stress and anxiety has been extensively studied but remains multifactorial (Borrow et al., 2016) and incompletely understood, the Research Topic of “*Stress, Anxiety, and the Synapse*” was proposed to generate a timely collection of articles that describe potential mechanisms through which stress could impact the expression of increased anxiety.

Bertholomey et al. confirm that control adult female rats display less anxiety-like behavior than males in an elevated plus maze. Chronic corticosterone (Cort) exposure, which partially mimics a chronic stress condition, had no impact on anxiety measures but increased immobility in the forced swim test, a behavioral marker of depression, in an age- and sex-specific manner. The investigators further identify impacts of Cort on the expression of AMPA receptor subunits GluA1 and GluA2/3 in the basolateral amygdala that may underlie the development of depression as opposed to anxiety. Combined with pharmacological manipulation in adults, their findings suggest that high AMPA receptor expression protects against maladaptive responses to stress. This work highlights the importance of evaluating anxiety and depression as distinct conditions that engage specific mechanisms in the brain.

Relatedly, Mallien et al. employ a GluA1 knockout strategy, Cort supplementation, or repeated stress induced by immobilization in young adult mice of both sexes. These approaches have been shown to induce a depressive phenotype, but differ in their severity. Similar to Bertholomey et al., this group found that GluA1 and Cort play critical roles in mediating behavioral responses to stressful stimuli. While anxiety-like behavior was not directly evaluated in this study, nest-building and burrowing behaviors were assessed as indicators of general wellbeing (Jirkof, 2014). GluA1-null male mice built nests with reduced complexity and showed an increased latency to incorporate new materials into their nest structure, suggesting diminished wellbeing. The same was true for some, but not all, of the female mice. Decreased burrowing was also observed following GluA1 gene deletion in both sexes, with a greater reduction in females compared to males. The Cort supplementation model also reduced burrowing and increased latency to integrate new nest material, but only in male mice. The varied responses by sex and stimulus strength observed in this study demonstrate the high degree of brain plasticity that underlies normal and disrupted behavior.

Early-life stress or adversity, as investigated by Hamdan et al. is known to increase the expression of anxiety-like behavior in adulthood. This was confirmed by open field and light-dark box testing which revealed enhanced measures of anxiety in adult male rats with a history of neonatal maternal separation (MatSep). Persistent changes at the level of the synapse induced by this paradigm included multiple indicators of reduced dopaminergic neurotransmission in animals exposed to MatSep, particularly in regions associated with anxiety such as the nucleus accumbens and the medial prefrontal cortex. Specifically, early-life stress led to increased expression of the synaptic proteins alpha-synuclein, dopamine transporter, and/or dopamine receptor-2 in these areas. MatSep did not affect the response to methamphetamine administration in a conditioned place preference paradigm, however, despite the predicted changes in dopamine signaling. These results suggest that altered neuronal communication as a consequence of chronic stress exposure can affect some behavioral outputs but not others.

In another contributing article, Maita et al. provide an excellent review of the role of the bed nucleus of the stria terminalis (BNST) in anxiety and in mediating responses to chronic stressors. Multiple models are considered, with a view toward understanding increased corticotropin-releasing factor (CRF) neuron excitability. Increased CRF secretion from the paraventricular hypothalamic nucleus (PVN) under conditions of chronic stress has long been known to occur. The anterior BNST is a regulator of the PVN and plays a critical role in behavioral responses under chronic stress, including increased anxiety. A new model of neuroplasticity is proposed, involving a stress-induced increase in the expression of type 2 CRF receptors in the oval subnucleus of the BNST (BNSTov). Binding of these receptors by CRF turns on the AMPA receptor and subsequent signaling pathways. Greater BNSTov activation enhances GABAergic inhibition of the anterodorsal BNST, which subsequently disinhibits PVN CRF neurons and facilitates more or continued CRF release. In line with other articles described herein, this review defines a role for the AMPA receptor in driving increased neuronal excitability and providing a mechanistic link between stress and the development of anxiety.

Collectively, the articles in this Research Topic give a wide view of chronic stress models, their impact on excitability in the brain, and their potential contribution to the development of increased anxiety or other conditions of impaired mental health. The importance of this type of research cannot be overstated, as global exposure to stressors continues to climb along with the burden of mental illness and its treatment or management. Varying results can be seen when stress is experienced early in development, during adolescence, or later in life. The severity and duration of a stressor also has important implications, as does the context in which the stress is experienced and the sex of the individual receiving the stress stimulus. Additional work remains to be done so that we may more fully understand the links between stress and anxiety, and the neural, endocrine, and other mechanisms that subserve them both.

## Author contributions

KG drafted the editorial, which was reviewed and edited by JF and DJ. All authors contributed to the article and approved the submitted version.

## Conflict of interest

The authors declare that the research was conducted in the absence of any commercial or financial relationships that could be construed as a potential conflict of interest.

## Publisher's note

All claims expressed in this article are solely those of the authors and do not necessarily represent those of their affiliated organizations, or those of the publisher, the editors and the reviewers. Any product that may be evaluated in this article, or claim that may be made by its manufacturer, is not guaranteed or endorsed by the publisher.
